# Accessing health services through the back door: a qualitative interview study investigating reasons why people participate in health research in Canada

**DOI:** 10.1186/1472-6939-14-40

**Published:** 2013-10-12

**Authors:** Anne Townsend, Susan M Cox

**Affiliations:** 1 Department of Occupational Science and Occupational Therapy, University of British Columbia, Wesbrook Mall, Vancouver, BC V6T 2B5, Canada; 2 Milan Ilich Arthritis Research Centre, 5591 No. 3 Road, Richmond, BC V6X 2C7, Canada; 3 W. Maurice Young Centre for Applied Ethics, School of Population and Public Health, University of British Columbia, 227-6356 Agricultural Rd, Vancouver, BC V6T 1Z2, Canada

**Keywords:** Human subjects protection, Health research, Therapeutic misconception, Self-managing, Qualitative research, Accessing health services, Informed consent, Recruitment

## Abstract

**Background:**

Although there is extensive information about why people participate in clinical trials, studies are largely based on quantitative evidence and typically focus on single conditions. Over the last decade investigations into why people volunteer for health research have become increasingly prominent across diverse research settings, offering variable based explanations of participation patterns driven primarily by recruitment concerns. Therapeutic misconception and altruism have emerged as predominant themes in this literature on motivations to participate in health research. This paper contributes to more recent qualitative approaches to understanding how and why people come to participate in various types of health research. We focus on the experience of participating and the meanings research participation has for people within the context of their lives and their health and illness biographies.

**Methods:**

This is a qualitative exploratory study informed by grounded theory strategies. Thirty-nine participants recruited in British Columbia and Manitoba, Canada, who had taken part in a diverse range of health research studies participated in semi-structured interviews. Participants described their experiences of health research participation including motivations for volunteering. Interviews were recorded, transcribed, and analyzed using constant comparisons. Coding and data management was supported by Nvivo-7.

**Results:**

A predominant theme to emerge was 'participation in health research to access health services.’ Participants described research as ways of accessing: (1) Medications that offered (hope of) relief; (2) better care; (3) technologies for monitoring health or illness. Participants perceived standard medical care to be a “trial and error” process akin to research, which further blurred the boundaries between research and treatment.

**Conclusions:**

Our findings have implications for recruitment, informed consent, and the dichotomizing of medical/health procedures as *either* research *or* treatment. Those with low health status may be more vulnerable to potential coercion, suggesting the need for a more cautious approach to obtaining consent. Our findings also indicate the need for boundary work in order to better differentiate treatment and research. It is important however to acknowledge a *categorical ambiguity*; it is not always the case that people are misinformed about the possible benefits of research procedures (i.e., therapeutic misconception); our participants were aware that the primary purpose of research is to gain new knowledge yet they also identified a range of actual health benefits arising from their participation.

## Background

In this paper we investigate how people come to take part in health research. Early studies in the medical based literature were driven primarily by recruitment concerns. Examining motivations to volunteer focused largely on disease specific clinical trials [1] and typically generated quantitative evidence that reported participation patterns [2]. Therapeutic misconception and altruism were framed as predominant reasons as to why people volunteered. Similarly, the bioethics and social science literatures have identified therapeutic misconception [3-8] and altruism as strongly associated with research participation [9-13].

Over the last decade investigations into why people volunteer for health research across diverse research designs have become increasingly prominent [10,14-17]. Studies have included a focus on the consent process in different contexts e.g. parental proxy consent for children in clinical trials [18] and patients’ perceptions of informed consent in cases of acute myocardial infarcation [19,20]. Limkakeng et al’s recent review of the literature about research participation in emergency medical conditions showed that among factors favouring participation, altruism and personal health benefit had the highest frequency [21]. Other studies have shown how people have reported volunteering for reasons of self-interest [22,23], personal preferences [24,25] the ease of research task e.g. nothing to lose in interview studies [26], and compensation; payment to healthy volunteers can be a strong incentive to participate in a range of studies [27]. Other motivating factors identified include the personal satisfaction of participants [28], aspects of the research relationship and the level and nature of information provided [29]. The crucial role of trust has also been discussed in a range of ways. For example participant trust (in the institution, the researcher or the research protection system) has been indicated as an important condition of taking part as opposed to a reason for taking part [30-33]. A recent study in India examining motivation for participation in non-therapeutic clinical trials found that for patients it was the request from the treating physician that was the main motivating factor (88%), while financial reward was the main reason for 65% of the healthy participants who also reported free medical check up/personal health gains as important reasons (43%) [34].

Increasingly evident is the complexity that underpins decisions to volunteer. Stunkel and Grady reviewed the literature on healthy volunteers for clinical trials and found that although financial reward was a strong motivator, it was not the sole reason to participate. They emphasized that individuals relayed multiple reasons to take part in clinical trials [35]. Fry and Dwyer [28] describe how motivations to participate in research may be better understood as 'multidimensional reasons’ that combine direct personal gains and benefits to others. Accordingly decisions to participate in research can rest on a combination of immediate and personal concerns, which may include practical considerations, levels of interest, the individual’s 'relational setting’ and more general values, which guide behaviors, for example, a sense of responsibility or a wish to contribute knowledge to the community [36].

'Clustered reasoning’ reveals how decisions to volunteer are often more nuanced and complex than suggested by models that identify single reasons. The clustered perspective emphasizes context and the relational aspects of research participation from the volunteer’s perspective. For example, Hallowell has highlighted how altruism can be tempered by the anticipation of burdensome research tasks [10]. Emerging evidence suggests that across research design from clinical trials to interviews, potential volunteers balance the (potentially erroneous) belief of benefits gained in terms of health care and/or personal interest/satisfaction with the anticipated burdens as well as risks of participation [10]. In this way, research participation is best understood as multidimensional where contextual features coalesce to enable, facilitate and motivate individuals to volunteer.

This paper contributes to more recent qualitative approaches to understanding why people volunteer for different types of health research. We undertook a contextual analysis [37] to explore the meanings health research participation holds for people in the context of their lives and health and illness biographies. We include diverse research designs to gain an understanding of wide-ranging experiences. We sought to understand the 'lived experience’ of participation, which has thus far been neglected in the literature. This approach recognizes a 'clustering’ of motivations, while seeking to identify personal priorities of volunteers. The varied health research designs in which our participants had been subjects, offered excellent opportunities to gain insight into the meanings, motivations and contexts of research participation. Here, we report on a predominant theme that emerged in our analysis; volunteering for health research in order to access health services in three ways: 1) Accessing medicines; 2) Accessing healthcare; 3) Accessing technologies for monitoring health.

## Methods

### Participants and recruitment

The research reported here was part of phase one of a six-year (2005–2011) three-phase study investigating human subjects’ experiences of health research participation. Our aim was to examine the perspectives of subjects and compare them with researcher and research ethics boards/institutional review boards (REB/IRB) members’ perceptions of how subjects experienced research participation. In order to gain multiple perspectives for comparative purposes, this phase entailed personal interviews across a broad range of human subjects, health researchers and REB/IRB members, scholars, and policy makers. Our findings are based on the interviews of the human subjects who participated in the phase one interviews. The project design has been described in more detail elsewhere together with analysis of other salient themes such as trust [33] and responsibility [36].

We recruited 41 participants (38 from British Columbia and 3 from Manitoba), who had volunteered to participate in one or more health research studies (23 women, 18 men) across multiple designs and areas (e.g. clinical, behavioral, public health). Participants lived in a mix of communities, with the majority living in or near large urban areas with access to hospitals and clinics in the vicinity. This range allowed us to make comparisons, and explore diverse experiences and perspectives. Our aim was to recruit approximately 40 human subjects. We did not anticipate theoretical saturation, but saturation of resources (e.g. time; data handling) [38]. We also adhered to suitable sampling size in grounded theory [39,40], and guidelines for in-depth analysis of a qualitative dataset [41]. Sampling was purposive [42] designed to yield maximum variation sampling. There was also some snowball sampling in that some participants told others about our study but this was not a deliberate strategy. This strategy allowed both an explicit comparison between criteria, and maximum variation in order to gain an understanding of participation. Because the rationale was to explore a heterogeneous sample of participants and identify diverse experiences, we employed multiple recruitment strategies: poster and media advertisements in diverse publications and health settings; opportunistic sampling (i.e. social and network connections), and consultations with relevant communities (such as seniors’ care facilities). Potential participants were invited to contact one of two research coordinators or the principal investigator (contact details on recruitment documents) if they were interested in participating in our study. One of the participants who contacted us did not take part in the scheduled interview because he could not be contacted subsequent to initial communications. Criteria for participant eligibility required that individuals were English speaking, had volunteered for one or more health research studies, were adults and were living in one of two Canadian provinces where the research was based, British Columbia or Manitoba. Individuals reported their health status at the time of their participation in our study as healthy (16), acutely ill (2), or chronically ill (23). Thirty-nine participants reported volunteering for one or more health studies that we grouped into clinical trials (20), behavioral studies (9), basic biomedical studies (7), and public health studies (3). Although most participants discussed taking part in more than one research study, we have classified them according to the health studies that were most salient in their discussions. (One participant not included in this tally described volunteering for but then not taking part in two research studies while another sought us out to describe his deceased father’s forced participation in military research). We audiotaped semi-structured interviews (37 face–to-face in non-clinical settings and 4 telephone) and a follow-up phone call for clarification as necessary (5 participants). Interviews were conducted in subjects’ homes, or at a place of their choosing (e.g. the university or a similar setting). The study was approved by Research Ethics Boards at: the University of British Columbia, Vancouver Coastal Health Authority, the University of Manitoba, the University of Winnipeg, St Boniface General Hospital Winnipeg. All participants gave written informed consent.

### Qualitative interviews

An open-ended semi-structured interview guide (see Additional file 1) was developed to elicit detailed accounts. The guide was formulated based on the literature review conducted prior to fieldwork and discussions involving the multi-disciplinary research team (including researchers from medical anthropology, sociology, law, philosophy and ethics). The guide was organized around six broad areas: 1) describing health research studies in which participants had been involved; 2) exploring participant motivations to volunteer in previous studies; 3) eliciting experiences of study participation; 4) probing what it means to be a human subject; 5) querying participants’ broader views about research participation human subjects; 6) asking about specific reasons for participating in our study. Probes and prompts were included in each section to encourage participants to reflect on their experiences and communicate their concerns and priorities in their own words. The audio-recorded interviews lasted an average of 70 minutes (ranging from 45-90 minutes).

### Analysis

The interviews were transcribed verbatim and checked against the recordings. Paper based methods, and the software package *NVivo 7* were used to manage data. Analysis was iterative, informed by grounded theory strategies [38]. We compared the transcripts to identify emergent themes (e.g. reciprocity) and also included *a priori* codes based on the interview guide. Three researchers read and annotated three transcripts independently with no predetermined coding structure. Initial codes were identified, discussed, clarified and agreed upon after negotiation. These codes included decision-making process; personal beliefs; reasons to participate; and impact of participation. Each researcher then coded the remaining transcripts, constantly comparing, discussing and amending codes. After further discussion, predominant themes were identified such as hope (for symptom relief and treatment); therapeutic (mis)conception (conceptualizing research as treatment); trust (research relationships). Consistency was looked for between and within transcripts, and deviant cases sought to increase trustworthiness of the analysis. The interview extracts reported in this paper reflect common concerns discussed by the participants either spontaneously or in response to an interviewer prompt or probe.

Because we sought to identify the practical circumstances surrounding the experience of volunteering as well as participants’ attitudes we draw on Fry’s 'phenomenology of participation’ [43]. Fry notes: “by examining the phenomenology of research participation—i.e. why and how people participate in research, and how and what they gain from this” we can learn more about volunteer motivation in the context of daily life and the research enterprise. In so doing we consider both the external and internal normative bases of research participation. We aimed to address the multi-dimensional factors involved in volunteering for research and sought to identify those aspects of daily life that became salient at particular times, focusing on the relational settings of individuals and their priorities. Following Fry [43] we sought to understand the context of the pragmatic and contextual dynamics of daily life and how values we live by (e.g. altruism) and institutional factors (e.g. availability of health services) are translated into normative behaviors (e.g. volunteering for research).

## Results

The participants’ age-range was estimated at n = 25 middle-aged (between 40–60) with n = 14 over 65 years and n = 2 unknown. Participants self-reported health status was: n = 23 chronic illness, n = 2 acute illness, and n = 16 healthy. Of the n = 41 participants recruited, this analysis is based on the accounts of n = 39 participants who described participating in a total of 141 health research studies between them. As described above, the remaining n = 2 participants had not taken part in research. Research participation ranged from 2 studies to 13; the average number of studies was 3–4 per participant. Most (n = 35) individuals reported 'clusters’ of factors motivating participation in health research (e.g. a mix of personal gain and practical contingencies and broader attitudes about opportunities to advance knowledge). A minority of participants (n = 4) identified payment as their prime motivator. These included a street involved person (n = 1); students (n = 2); and an unemployed person with a need for 'pocket money’ (n = 1). Others who mentioned compensation described financial reward as insignificant in their decision to participate (n = 15). Of those who took part in surveys and interviews, several described the combination of ease of research task, their personal interest and making a contribution to knowledge as motivating them. More than a third (n = 15) conveyed either being a good citizen or a volunteer as an important part of their identity, although only one participant noted this as an explicit motivator. Nearly all (n = 16 of n = 20) of the participants who discussed clinical drug trials hoped for symptomatic relief or some other kind of health benefit (these included drug trials for participants with cancer, HIV and other chronic conditions such as arthritis). Only 2 who took part in clinical drug trials identified as healthy at the time of their trial participation. Both had taken part in a herpes drug trial; one had done so many years before and the payment of $500 was the prime motivator; the other participant noted her interest in research combined with the fact that she had been a patient at the clinic and trusted the team, as her motivations. Only one person who had taken part in a clinical trial relating to his illness condition (Chronic obstructive pulmonary disease - COPD), which tested the interactions of a 'flu shot’ with COPD, reported explicitly that he did not volunteer for health benefits. He described how he had been a volunteer throughout his life, and now at the age of 78, he had an interest in research, and participating was a way he could continue to volunteer, an identity with which he strongly identified. Another participant with diabetes described participating in a clinical trial (testing medications) because he was keen to contribute to 'the cause’ of diabetes, was interested in research and out of concern for his grandchildren, rather than for personal health or treatment gains.

A predominant theme to emerge from the data was how people volunteered for health research in order to access health services or health information (for themselves and/or family members). Twenty-five of the participants reported that they took part in studies primarily for health gains, either because they had been diagnosed with chronic or acute conditions, or because they saw participation as a way of monitoring their health and a means to prevent illness developing. Here we report on the three predominant ways participants described volunteering for research as a way of accessing health benefits. All themes described below emerged in an inductive manner during the process of data analysis.

### Accessing medicines through clinical trials: “a way of dealing with the disease”

Of the n = 20 participants who discussed in detail participating in clinical trials, n = 16 reported that they volunteered primarily to access treatment. Entering a drug trial meant acquiring medication that was unavailable to them as patients. Many described a 'process of trial and error’ with prescribed drugs as they sought symptom/disease control as patients. Most clinical drug trial participants expressed their hope for gaining effective treatment, while contributing to research.

People volunteered for studies in their ongoing attempts to manage illness, relieve their symptoms and control their disease. Some reported being recruited by a trusted physician (general practitioner or specialist), who they perceived would not put them at risk of harm via research. Cost was an added factor. One participant with severe and debilitating osteoarthritis participated in a Vioxx (Rofecoxib) (post-marketing Phase Four) drug trial primarily in the hope of symptom relief and a way of dealing with the disease; her symptoms had proved resistant to prescribed medications. She described how trust in her doctor and an inability to pay for efficacious medications were also factors in her decision:

*My doctor asked me [to participate]… She said this drug was really good… she could start me on this drug… I guess she just thought that it might be helpful to me… I said, “Sure”…. I trusted her. … What I was using at the time wasn’t working very well, … That way all the drugs would be paid for, which was GREAT… I was having a hard time getting around, so I thought, well maybe this will work… to alleviate the pain…. a very expensive drug… couldn’t have afforded it… not covered by Pharmacare… risks reasonable.… hard to be this debilitated… I have to deal with it.* (813, arthritis, phase 4 clinical trial).

This quote illustrates research participation as a way of overcoming a financial barrier (the cost of the medication) to potentially effective medications. Vioxx (Rofecoxib) via the clinical trial provided much sought relief, and facilitated a return to this participant’s active life and paid employment – all conveyed as major benefits.

Another participant pursued ongoing medical tests, a series of different treatments and experienced adverse reactions as she sought relief via health care. In this context, she participated in the hope of gaining symptom relief:

*… the respiratory (clinical trial) one, I was asked… if I’d be willing to participate, by a doctor I was being treated by… He said: “There’s absolutely no pressure”… I’d had ALL those tests… for years… I wouldn’t mind participating, 'coz I’d already… done tests… I trusted my respirologist…. I just went on faith that if my doctor had asked me to participate, and he knew about the study, and he knew the doctor that was running the study… everything would be fine* (819, chronic cough, clinical trial).

The aim of gaining effective treatment motivated this participant to volunteer, while her faith in her doctor’s knowledge of the study increased her level of comfort in participating. This participant was clear about the difference between research and treatment, but for her, medical treatment involved a series of “different medications” and “every test done under the sun”, thus her experiences of research and treatment were similarly experimental, i.e. both essentially a process of 'trial and error.’

Illness and treatment trajectories figured signifcantly in decisions to participate in clinical trials. One participant, newly diagnosed with rheumatoid arthritis conveys how issues of risk changed according to her need for effective treatment:

*I was not only sick, I was having to get my head around taking noxious chemicals… for the rest of my life… knowing that I was no longer able to work… the number of changes that occurred were catastrophic… so I was dealing with those as well. NOW, my rheumatologist… presented me with the opportunity to participate in this trial, and my reaction to that was, “I’m having to take all of the pills that I’m taking now, plus I’m trying to get my head around this, plus I’m trying to help my family get their heads around this, and you’re asking me to swallow something which is an unknown chemical? I don’t think so!”… Four months later, I just continued to get worse… I went back to him, and said, “Is this still available?” And he said, “Yes, we have one more opening”* (207, rheumatoid arthritis, clinical trial).

This participant went on to describe how the trial medications controlled her symptoms and offered relief that was unavailable to her via regular health care.

### Accessing health care through research participation: “feeling well cared for”

Some participants volunteered for research to access care that was either unavailable to them or difficult to access in the health care system. One individual who had multiple health issues and took a range of medications which required monitoring, participated in several studies seeking the care he required for his complex needs: *“I had to go through research to get proper care, to get the BEST care, it shouldn’t be the way it is, but it often is* (202, HIV positive, clinical trials). Another participant in a drug trial for cholesterol management described feeling 'cared for’ during the research:

*Positive things about being in the study; I remember feeling very well-cared for, because it was like such a regimented, regular visits… the regular appointment healthcare system, you make your appointment to go to the doctor, you wait forever if you have to go to see a specialist…”* (815, clinical trial, family history of cholesterol problems).

Others who were diagnosed with conditions that required various medical treatments as part of their care, welcomed research participation to access procedures and knowledge that was otherwise unavailable to them. HIV and cancer patients emphasized this point. One participant in a study monitoring the effects of HIV medications on bone loss reported health benefits of participation:

*I… was very interested in osteoporosis as a personal trainer… and then I was getting a free scan for my whole body for osteoporosis… so it was better care. … the results of that, I was able to find out really quickly. They showed me all of my stuff, which you don’t always get, my bone scans… and my own doctor got all that. He was able to share all that with me. Now that’s a huge benefit that people don’t often get, is to be able to see ALL the results* (202, HIV, clinical trials).

Research offered this participant, as an informed and active patient, a way of managing his disease. He gained more comprehensive care, including more time with health care providers during the study and increased, pertinent personalized information via access to his medical test results.

### Accessing research technologies for monitoring health: “getting to know your situation”

Accessing technologies offers possibilities for monitoring health and obtaining early diagnosis that can prevent or limit disease and therefore has important ramifications for future health and self-care. One participant emphasized gaining cost-free knowledge in noting the benefits to screening (e.g. blood tests or a PAP smear): “*You’re up to speed with all sorts of your latest medical exams”* (823, healthy, various screenings, multiple studies). This participant considered there was no downside risk; on learning the results, she would know her health status, and either enjoy the relief of knowing all was well or be alerted to the need to take action for optimum effect. In the current context of self-care, self-management and individual responsibility, monitoring becomes central to maintaining good health and preventing disease:

*… I smoked for thirty years… my concerns were my lungs, so… it would reassure me or not reassure me whether I was, healthy or not … one of the things… it DID bring… is that I had a scar on my lung… I had to go for CT scans every six months for about two years to see that it wasn’t cancerous. And it wasn’t… so they did a lung… bronchoscopy… I had a lot of lung tests… which I thought was very beneficial, because… you go to the doctor 'n they’ll say: “Oh, you have a chest,” or whatever … I just felt like doctors minimize a lot of things and this was an opportunity for me to just say, “Okay, now I feel better,” or “Let’s deal with it”…* (816, healthy ex-smoker, lung health assessment).

Other accounts revealed how the family doctor or specialist sometimes occupied a central role in research recruitment:

*… it was just my cancer doctor out of the blue sent me on this PET scan –…’advancing beyond an MRI’…. I would never have volunteered you don’t seek it out…. he wasn’t happy about a lump on my palate. (Two days later) I got a call from (the clinic receptionist): She said: “I’m from the PET centre”, and I thought I don’t have a pet’…’* (315, cancer, monitoring disease via PET scan)

The doctor’s role was highlighted by the participant’s phrase 'out of the blue,’ and miscommunication about the PET scan. He explained that after discussion (with the receptionist) he participated: “… *for my health… I did because it was part of my cancer”* (315, cancer, monitoring disease).

Others sought free examinations to monitor general health. For those who reported being healthy and had no known high risks due to lifestyle or other factors, research participation provided a means of securing a health check-up at no cost^a^. As one participant indicated, the anticipated and actual benefits were twofold:

*Now one of the benefits often that these have is that you’re up to speed with all sorts of your latest medical exam. If in a screening process they take blood, even if they’re not looking for this, if they see something, you’ll be advised. And same with a urine test and same with a PAP test, or any kind of test.… you get these exams for free, and you get to know what’s your situation* (823, healthy, multiple studies, various screenings).

Research participation was a way of securing specialist care sooner than it would have otherwise been available as well as accessing regular appointments. In such examples gaining better care, as the participants perceived it, was a direct, major benefit. This illustrates how active and informed patients, keen to engage in self-care and monitor their condition, play an important role in health research. Another participant described how entering research gave her information and peace of mind:

*… so… when they did… the ultrasound of my arteries and… reported that there was no plaque build-up I remember thinking “I’m really lucky to be in this study, and have this done“. Because that’s always something that’s in the back of my mind that I’m gonna die of a heart attack, and my arteries are all plugged… so to hear that … I felt like euphoric… and I never would’ve had that test outside the study* (815, clinical trial, family history of cholesterol problems).

This woman self reported as 'healthy’ with a family history of heart problems. Although clearly understanding that she was participating in research (a drug trial), she also saw the research as health care, in the context of what she described as 'impersonal and inflexible care from her GP’. The concrete benefits arising from participation included gaining new diagnostic information, which eased her prior anxieties about heart disease.

Altruism also featured in the accounts of accessing health treatments and technologies. For the majority of participants, however, altruism was not a prime motivator. Rather, the participants expressed altruism alongside other determining factors such as hope. For example several were driven by the hope of gaining personal health benefits while also hoping that their actions (e.g. participation in a clinical trial) would benefit family members or others with the same condition. In this way participants’ motivations were both self and other centred:

*I suffer from familial-hypercholesterolemia… I suppose that I’m just so keen to have them discover something that would be a viable treatment that I’m willing to take the risks involved, for myself and for others who are in the same circumstances…* (810, cholesterol, clinical trial).

## Discussion

Analysis of the 39 in-depth interviews illuminates a core set of motivating factors that help to explain why many people volunteer for health research. Our findings provide a contextual understanding, beyond the point of decision at informed consent, of the meanings research participation holds for individuals in the context of their lives. Extending beyond motivators of trust, therapeutic misconception, and altruism we report participant priorities in terms of personal health, help-seeking and self-management actions. Our analysis reveals the individual context of daily life, and health, illness and treatment trajectories, against the broader backdrop of the health care system and notions of the active patient who is encouraged to self-manage. Thus, these findings prompt reflection on access issues. If participants are participating in research, such as clinical trials to gain access to services and care, this has implications for researchers and the consent process, but it is also a (somewhat negative) commentary on how we provide access to healthcare (or fail to).

For many of our participants, the direct benefit of accessing treatment, care and/or information was a prime motivator for volunteering in health research. While factors such as trust were important, pragmatic concerns played a greater role in shaping action. The accounts featured here also highlight the contextual significance of seeking care in an overburdened health care system.

Participant accounts included a range of research designs, in the context of diverse lives, and health and illness circumstances. Participant experiences however, were anchored in broadly shared cultural and structural systems. For example, accounts illustrated common values such as responsibility (in terms of a commitment to honest and sincere participation in the research) [36] and altruism (which reportedly influenced but typically did not drive actions). Institutional values included trust in health care systems, and support for the pursuit of knowledge and the scientific endeavor [33]. Those who took part in behavioral studies typically considered the research tasks and participation to be relatively innocuous or non-intrusive (e.g., surveys, interviews); for some they were an opportunity to satisfy an interest or share their experience. Participation was influenced by the practical circumstances of daily life and the perceived burden or convenience of research tasks. Most prominent in the accounts however, was the framing of research participation as a way of gaining direct benefits of accessing health services that were otherwise unavailable or difficult to obtain (treatments, technological monitoring, more care, gaining health information) [44]. Research participation for many of our participants *offered access to* treatment and was part of a help-seeking process in lives fraught with illness, occupied by the search for symptom relief or the hope of prolonging life. There has been some evidence in studies of this phenomenon. For example, others have shown how research participation in emergency settings is regarded as having direct health benefits, in some cases over and above what the health care system can offer [19-21,45,46]. Our analysis also revealed how people used research as a strategy to self-manage their health conditions. Thus, it would be a mistake to broadly construe their motivations to participate in health research as a form of therapeutic misconception, “the notion that unless otherwise informed, research subjects will assume (especially, but not exclusively, in therapeutic research) that decisions about their care are being made solely with their benefit in mind” [3] Our participants appeared to have no such illusions. Thus it may be appropriate to coin a new phrase for this, that of 'therapeutic research participation”. Our participants seemed to be well aware of the risks they faced, the uncertainty of direct therapeutic benefit and the limits of research. In several cases participants very clearly indicated that they sought the ancillaries of research: human contact; routine; observation and testing. This is important because it positions participants as patients, as well informed, actively help-seeking, self-caring (taking broad actions for health) and self-managing (e.g. managing symptoms through access to proven effective medications).

Various studies investigating research participation have identified how participants’ accounts portray complex and layered experiences overall and blurred boundaries between patient and participant, physician and researcher and treatment and research [44,47]. Hallowell and colleagues [47] identify how patients find it difficult to differentiate genetic testing from care and how clinical research participants conflate these activities. We found something different. Our participants did not conflate or fail to differentiate between research and care [48]. But, they accessed treatment, care and screening (health information) through research, while being quite aware that the research (for many of them like healthcare) does not offer direct promise of therapeutic benefit. There was some evidence of this in Gammelgaard et al’s [19] research whereby at least some of the participants identified how they felt they could access more sophisticated technology which would be of greater benefits to them in emergency situations, at the same time recognizing that they were participating in research (although many were confused about whether the procedures they had agreed to were research or treatment).

In particular, people with chronic conditions have an established and ongoing biography of illness [49]. This involves accessing healthcare and help seeking for symptom control or to limit the impact and course of disease [50]. In this context, for the majority of participants in our study, health research participation was an integral part of overall self-management and help-seeking experience [44]. This blurring of research and treatment is underlined when we consider how patients with multiple morbidities are prescribed complex drug regimens and suffer adverse effects in a (drug) 'trial and error’ process as their physician 'experiments’ with their treatment program [51]. In this way, people with life threatening or complex illness conditions often experience health services care *as* experimental.

It is important to note that when people participate in health research to access health services, tensions emerge. In a system that dichotomizes research and treatment, contradictions arise if participants view research as research *and* as treatment; the two are not mutually exclusive, but inextricably bound. If these 'categorical ambiguities’ [47] are surfaced we can gain more insight into the 'lived experience’ of research participation. In highlighting what amount to, for some, 'indivisible distinctions between research and treatment’ significant questions are raised about: the extent to which this occurs and how far health professionals perceive research as treatment. It also prompts discussion about how far the distinction between research and treatment can be sharply divided, in that medicine is not an exact science, and experimental trial and error, as well as risks and benefits, far from being the province of research is widespread in the practice of medicine.

The implications of our findings then are not just relevant to understanding the motivations of volunteers. The findings shed light on what may be a serious flaw in the intersection of health care and health research. If potential participants rightly or wrongly believe that access to normal care, information or treatment can be fast-tracked through a kind of Nexus line of research participation, then something has truly gone wrong with health services delivery and provision. For the research enterprise it suggests a kind of subtle coercion of participants in that whether or not they gain health benefits, they are open to risk in a way that they would not be were they able to readily access standard procedures. We are well aware that standard wording in consent forms is meant to exclude this possibility. However, there are worrisome indications in our data that participants see the situation differently and it may well be that policy-makers are blinding themselves to current realities, and that the quality of human subjects protection is compromised [52].

### Study limitations

We did not include non-participants in our study, and so were unable to investigate this perspective, which would no doubt offer additional insight into salient factors or circumstances that mitigate against as well as promote research participation. Although some people expressed hypothetical aversions to participation, in clinical trials for example, we cannot be sure that in real life circumstances they would decline. The topic of research participation may have meant that those who had a particular story to tell or a particular interest in research were more likely to participate. The context of giving accounts in research may be problematic too as people tend to present positive identities in interviews by drawing on cultural assumptions about appropriate behaviors. This may influence how people frame their participation in terms of, for example, altruism or logical motivations. We did not circulate our findings to participants to cross check whether they might have identified discrepancies with our interpretation although we did, during the interviews, confirm emergent understandings and summarize them at the conclusion. Also, we have no record of the patient physician conversations and how recruitment unfolded. The detailed accounts and iterative and comparative analysis however, offered insights into experiences of how people came to take part in a range of health studies, in the context of their illness experience, and the perceived benefits of the research in which they participated.

## Conclusion

Our findings have implications for recruitment, informed consent, and the dichotomizing of medical/health procedures as *either* research *or* treatment. Health status influenced how individuals perceived risks and benefits, and trust in research. This has a bearing on vulnerability and potential coercion, which could include 'deliberate or inadvertent exploitation of vulnerability’ during informed consent for clinical drug trial participation, particularly for the profoundly ill. This calls for a more cautious consent process. Potential participants need to recognise that they are giving consent for something (e.g. a procedure) that may offer treatment, better care, or diagnostic possibilities in addition to research participation, but health professionals (in theory) and the formalized system clearly differentiate these. This poses problems for researchers who need to understand that although participants may fully understand the aim of a procedure is to gain research data, they may be motivated to volunteer in the hope that participation will make their life better, or longer. This is consistent with the need for boundary work in order to demarcate the difference between treatment and research, but also to acknowledge that there is *categorical ambiguity*. Our findings highlight that while many participants may recognize research *as* research they may still participate in order to access (otherwise unavailable) health services in a range of forms. Accessing health services through research participation also offers a different perspective on the concept of therapeutic misconception by highlighting systemic inequities in health delivery as a fundamental condition shaping the desire to participate in research. An important structural issue is also raised about how the context of research recruitment is shaped. It also poses the question: Are the most vulnerable and those with the fewest health care alternatives taking substantially greater risks than (and for) the rest of the population? If research is a means of accessing treatment, we must be concerned about the context in which decisions about research participation are made and the potential that this has for skewing participant sampling and the outcomes of health research, including clinical trials. Caution must be advocated when attempting to establish participants’ motives for becoming involved in different types of research and greater attention must be given to ascertaining the consequences of seeking treatment, care or health information through the 'back door’ of research participation.

## Endnotes

^a^The Medical Services Plan (MSP) insures medically required services provided by physicians and supplementary health care practitioners, laboratory services and diagnostic procedures. In British Columbia best practice for physicians is not to routinely provide annual physical exams unless the physician determines they are in the patients’ best interests.

## Abbreviations

COPD: Chronic obstructive pulmonary disease.

## Competing interests

The authors declare that they have no competing interests.

## Authors’ contributions

AT undertook some of the interviews, lead the specific thematic analysis and interpretation of the data set and the writing of all drafts. SC undertook a large proportion of the interviews, analyzed and interpreted the data, and contributed to all drafts of the paper. Both authors read and approved the final manuscript.

## Authors’ information

AT is a medical sociologist and research associate at the University of British Columbia (UBC). AT is a Co-investigator on the project. SC is an Associate Professor at the Maurice young Centre for Applied Ethics (UBC). SC is the lead Principal Investigator on the project.

## Pre-publication history

The pre-publication history for this paper can be accessed here:

http://www.biomedcentral.com/1472-6939/14/40/prepub

## Supplementary Material

Additional file 1 Interview guide. Click here for file
