# Supplementary material for: Accessing health services through the back door: a qualitative interview study investigating reasons why people participate in health research in Canada
Source: BMC Med Ethics. 2013 Oct 12;14:40. doi: 10.1186/1472-6939-14-40 (PMC3853104; doi:10.1186/1472-6939-14-40)
Supplement: Additional file 1 — Interview guide. [file 1472-6939-14-40-S1.doc]

# **Phase I Interview topics for human subjects (with suggested probes)**

**Discussion and Signing of the Consent Form:**

**With your permission, I would like to record our preliminary discussion about the study and the information in the consent form provided to you.**

- Check participant has had time to read consent form.
- Review essential aspects (i.e., purpose of study, what is being asked, confidentiality, how we will handle any concerns, risk and agreement to maintain data for longer than normal)
- Ask if there are any questions or concerns.
- Double check that all required boxes are checked and that participant has a signed copy of the consent form for their records.

Start of Interview Questions (Recorded):

Please tell me a little about the research study (or studies) you were involved in.

- How would you describe the purpose of the research?
- How recently were you involved in this study?
- What was being asked of you as a research participant?

**Please tell me about your decision to become involved in the research.**

- Where did you learn about the research?
- Did you have a particular interest in the topic of the research? (Probe for interests in particular health conditions, family history, employment in health care sector)
- How did you decide to participate?
- Was there anyone in particular who was helpful to you in making this decision? How were they helpful?
- Did you feel any opposition to or pressure to participate in the research? From the researchers? A health care provider? A family member or friend?
- Do you feel you were provided with enough information about the research? And about the researchers?
- Do you feel you had an adequate understanding of any risks/benefits associated with your participation in the research?

**Please tell me about your experience when you were involved in the research.**

- What did you have to do as a research participant?
- How often was this asked of you? And by whom?
- Were there any unexpected requests or surprises?
- Was there any compensation for your participation in the research?
- Do you feel you were treated ethically? Why or why not? (Probe for meaning of “ethically” here. If unclear, ask about respect, privacy, provision of information about study, etc)
- Did you trust the researcher(s)? Did you have confidence in their ability to safeguard your privacy?
- Did you have any concerns about what was being asked of you?

Please describe what the experience of being a research participant means to you.

- Is there a responsibility to participate in research? Why or why not?
- Do you think your experiences have changed the way you think about research at all? For the better? Or worse?
- Do you now have any special thoughts and/or regrets about participating in the research?
- Did you learn about the findings of the research you participated in?
- Will you consider participating in another research study? Why or why not?

If I could broaden the discussion, I would like to hear your views on how research participants are regarded and/or treated.

- Do you feel there is adequate protection/recognition for research participants? Why or why not?
- What do you think research participants have a right to expect in return for participation in research?
- Is there anything you would recommend to improve the situation of research participants? (Could refer to news stories about research ethics if appropriate)

To conclude, it would be helpful to hear your thoughts on this study.

- Why did you decide to participate in this study?
- What do you hope we will learn from this study?
- Do you have any other comments, suggestions or perhaps questions you think we should ask of other participants?

**Thank you for your generous participation in our study.**

**Check before leaving:**

- Completed consent form
- Permission to re-contact for clarification of issues arising in this interview and to ask permission to see if interested in future participation in this study (e.g., focus group)
- Complete mailing address
